# Domains of quality of life freely expressed by cancer patients and their caregivers: contribution of the SEIQoL

**DOI:** 10.1186/s12955-017-0672-2

**Published:** 2017-05-12

**Authors:** Zeinab Hamidou, Karine Baumstarck, Olivier Chinot, Fabrice Barlesi, Sébastien Salas, Tanguy Leroy, Pascal Auquier

**Affiliations:** 1 EA3279, Self-perceived Health Assessment Research Unit, Aix Marseille Université, 27 bd Jean Moulin, Marseille, cedex 05, F-13385 France; 2 National Clinical research Quality of Life in Oncology Platform, Nancy, France; 3 Department of Neuro-Oncology, Assistance Publique Hôpitaux de Marseille, Timone Hospital, 13005 Marseille, France; 4 Assistance Publique Hôpitaux de Marseille, Multidisciplinary Oncology and Therapeutic Innovations Department, Aix Marseille University, Centre d’Investigation Clinique, 13385 Marseille, France; 5 Department of adult oncology, Assistance Publique Hôpitaux de Marseille, Timone Hospital, 13005 Marseille, and CRO2, Aix Marseille Université, 13284 Marseille, France; 6 Université Lumière Lyon 2, Social Psychology Research Group (GRePS EA 4163), Bron, France

## Abstract

**Background:**

The purposes of this study, performed on a large sample of cancer patient-caregiver dyads, were: i) to simultaneously investigate, using an individualized quality of life (QoL) measure (Schedule for the Evaluation of Individual QoL, SEIQoL), the QoL domains freely expressed by cancer patients and their caregivers, and ii) to explore overlapping between the SEIQoL assessment and QoL assessment using traditional instruments.

**Methods:**

The study employed a cross-sectional design including cancer patients who were going to receive chemotherapy treatment and their caregivers. Quality of life was assessed using condition-specific questionnaires (EORTC QLQ-C30 and CarGOQoL), generic health-related questionnaire (SF-36), and open individualized measure (SEIQoL).

**Results:**

The final sample included 205 patient-caregiver dyads. From the SEIQoL, Family, Health, and Leisures were the most freely expressed QoL domains by patients and caregivers, but reported with different weights. Love life and financial issues were less spontaneously mentioned. The SEIQoL index was moderately correlated to the condition-specific QoL questionnaires (R lower than |0.40|) and to SF-36 (correlation coefficients: R ranging from 0.17 to 0.31).

**Conclusion:**

Individualized QoL measures allow individuals to spontaneously express important, non-predefined domains. This study highlights the need to explore QoL using a combination of individualized questionnaires and standardized questionnaires, capturing complementary facets that patients consider important in their life.

## Background

Studies examining health-related quality of life (QoL) have typically used standardized measures, called disease- or condition-specific, or generic measures [1]. These traditional standardized instruments usually include a pre-defined set of items exploring pre-defined domains and have been criticized for potentially missing important domains for assessing an individual’s QoL or including less important domains. To overcome this limitation, individualized measures or patient-generated QoL procedures have been developed, enabling respondents to spontaneously identify which aspects of their lives are actually relevant to their QoL. Thus, individual QoL assessment does not impose any predetermined external value system regarding either the choice of relevant domains or their actual significance for people [2]. This approach seems more suitable than standardized measures in capturing a patient’s actual perspectives [3].

During the last decade, an individualized open QoL measure has been developed, the Schedule for the Evaluation of Individual QoL - Direct Weighting (SEIQoL-DW), which was derived from the original SEIQoL [4–6]. The SEIQoL-DW assesses the individuals’ perception of his or her present QoL and allows the individual to choose the domains to be evaluated followed by a weighting procedure resulting in qualitative and quantitative outcomes. The SEIQoL-DW involves a semi-structured interview to enable individuals to spontaneously and freely name areas that appear important in life. The SEIQoL-DW has been reported as a feasible and valid instrument used in quantitative research [2, 7–9] and outside the research setting [10], with a limited burden on participants [3]. The SEIQoL-DW has proven to be a reliable measure in various populations [3].

SEIQoL-DW has been validated among numerous sick and healthy populations [6, 8, 11, 12], including patients with severe illnesses [2, 13]. The SEIQoL-DW has also been used as the primary outcome in recent large randomized controlled trials focusing on palliative care [14] and multiple sclerosis patients [15]. While the use of the SEIQoL-DW is recognized, few studies have provided SEIQoL-DW data for specific caregivers populations [16, 17]; to our knowledge, only one study, performed on amyotrophic lateral sclerosis patients, simultaneously provided the QoL of caregivers and patients using SEIQoL-DW [18, 19]. Moreover, few studies have explored links between QoL assessment using traditional standardized instruments and individualized measures, neither among patients nor caregivers.

In this context, the purposes of the present study, performed on a large sample of cancer patient-caregiver dyads, were to: i) simultaneously investigate, using the SEIQoL-DW, the QoL domains freely expressed by cancer patients and their caregivers, and ii) explore overlapping between the SEIQoL and traditional instruments.

## Methods

### Setting

The present study employed a cross-sectional design including cancer patients administered chemotherapy treatment and their caregivers from two French Public Academic Teaching Hospitals (the Oncology and Therapeutic Innovations Department, North Hospital, Marseille, and the Neuro-Oncology Department, Timone Hospital, Marseille). The participating oncologic departments treated patients presenting predominantly lung cancer, prostate/urologic cancer, breast cancer, and genital cancer.

### Population

The population included patient-caregiver dyads. The following inclusion and exclusion criteria of the dyads were considered:

- Patients: individuals 18 years of age or older, having a cancer defined by histology whatever the origin of the primitive, loco-regionally advanced or metastatic cancer, with an indication of chemotherapy, and able to speak/read French.
- Caregivers: individuals 18 years of age or older, designated as the primary caregiver by the patient, able to speak/read French, and free from cancer comorbidity.

### Ethics approval and consent to participate

All patients were fully informed of the study and provided written informed consent. The protocol was approved by the ethics committees of Aix-Marseille University, France (reference number: 2014-09-30-05).

### Data collection and measures

The data for age, gender, educational level, marital status, and professional status were collected for both patient and caregiver. The relationship between the patient and the caregiver was determined: partners, children/parents, brother/sister, and others. For the patient, the localization of cancer, the WHO performance status, and the presence of nodes/metastasis were assessed.

### Quality of life

Specific QoL questionnaires were administered: the European Organization for Research and Treatment of Cancer quality of life questionnaire (EORTC QLQ-C30) for patients, and the CareGiver Oncology Quality of Life questionnaire (CarGOQoL) for caregivers. Generic QoL questionnaires were administered to patients and caregivers: the SEIQoL and the Short Form 36 (SF-36).

The EORTC QLQ-C30 is a disease-specific questionnaire assessing the QoL of cancer patients [20]. It includes 30 items describing five functional scales (physical, role, emotional, cognitive, and social), nine symptom scales and single symptom items, and a global health status scale. The scores range from 0 to 100 for each scales/item. A high score for a functional scale represents a high/healthy level of functioning, a high score for the global health status scale represents a high QoL, and a high score for a symptom scale represents a high level of symptomatology.

The CarGOQoL is a well-validated condition-specific questionnaire for caregivers of cancer patients [21]. It includes 29 items describing 10 dimensions: psychological well-being, burden, relationship with health care, administration and finances, coping, physical wellbeing, self-esteem, leisure time, social support and private life. An index was computed. All dimension scores and the index are indicated on a 0–100 scale. A higher score indicates a better QoL.

The SF-36 is a generic health-related QoL questionnaire [22], including 36 items describing eight subscales (Physical function, Social functioning, Role physical, Role emotional, Mental health, Vitality, Body pain and General health), yielding two component summary measures (physical and mental composite scores, PCS-SF36 and MCS-SF36). The SF-36 scoring ranges from 0 (lowest) to 100 (highest QoL level).

The procedure of QoL assessment using the SEIQoL-DW involves a semi-structured interview and follows 3 steps. First the participant is invited to nominate the ‘5 domains’ he/she currently consider to be the most important in his/her life at the time of the assessment. If he/she finds it difficult to nominate the 5 domains, then a standard list of prompts is proposed. Second, the participant is also asked to rate the importance of each nominated domain on a visual analogue scale from 0 (lowest) to 100 (highest importance): the SEIQoL-VAS. Third the participant is asked for the relative importance of each area by a weighting procedure; the participant should quantify the relative importance of each of the 5 nominated areas, where all areas add up to 100: the SEIQoL-weight. The initial patient’s information was transcribed and analyzed by a psychologist (TL) experienced in categorizing patient comments into major themes. He aggregated the original information into 8 mains domains (cues) based on similar meanings. An overall index was obtained by summing the products of the rating (SEIQoL-VAS) with the weight (SEIQoL-weight) of each domain.

### Data analysis

The number of patients and caregivers with expressed domains from the SEIQoL were counted. For each domain, the median scores (raw and weighted scores) were provided for patients and caregivers, and the proportions of dyads (patient and caregiver) expressing these domains were provided. For dyads with common domains expressed by both patient and caregiver, scores and weights were compared using Wilcoxon tests. Spearman’s correlations between SEIQoL index and other standardized QoL scores (SF-36, EORTC QLQ-C30, and CarGOQoL) were provided.

## Results

### Characteristics of patient–caregivers dyads

Among the 388 cancer patients participating in the present study, 205 individuals who completed the SEIQoL-DW were able to nominate a caregiver agreeing to participate. Therefore, the final sample in the present study comprised 205 patient-caregiver dyads. For patients, the mean time since diagnosis was 39 days (Standard deviation 35.1). Approximately, 28% of the patients were diagnosed with lung cancer, 20% of the patients were diagnosed with gynecologic cancer and 10% of the patients were diagnosed with urologic cancer. The relationships between the patients and caregivers were partner relations for 61% of cases. All patient and caregiver characteristics are presented in Table 1.

**Table 1:** Main characteristics of the 205 patient-caregiver dyads

		N (%)	MD
		M ± SD	%
1. Patients			
Age	Years	61.5 ± 11.7	7.8
Gender	Woman	91 (44.4)	0
	Man	114 (55.6)	
Educational level	<12 years	108 (58.1)	9.3
	> = 12 years	78 (41.9)	
Marital status	Single	51 (26.2)	4.9
	Couple	144 (73.8)	
Professional status	Worker	55 (33.1)	19.0
	Not worker	111 (66.9)	
Localization of cancer	Lung	56 (28.3)	3.4
	Head and neck	51 (24.8)	
	Gynecologic	39 (19.7)	
	Urologic	21 (10.6)	
	Soft tissue sarcoma	8	
	Digestive	7	
	Dermatologic	6	
	Lymphoma	3	
	Bone	3	
	Adrenal	2	
	Undefined	2	
WHO Performans status	0	125 (63.5)	3.9
	> = 1	72 (36.5)	
Nodes		109 (64.5)	17.6
Metastasis		67 (36.2)	9.8
Time since diagnosis	Days	39.08 ± 35.1	9.8
2. Caregivers			
Age	Years	55,6 ± 14,4	22.4
Relationship with the patient	Partner	125 (61.0)	0
	Children	44 (21.5)	
	Brother/sister	12 (5.9)	
	Parent	6 (2.9)	
	Others^a^	18 (8.8)	
Gender	Woman	146 (71.2)	0
	Man	59 (28.8)	
Educational level	<12 years	79 (48.8)	21.0
	> = 12 years	83 (51.2)	
Marital status	Single	30 (15.8)	7.3
	Couple	160 (84.2)	
Professional status	Worker	31 (16.4)	7.8
	Not worker	158 (83.6)	

^a^friend, other member of the family, previous partner, other cases

N (%): effective (percents); M ± SD: mean ± standard deviation; MD: missing data

### SEIQoL domains freely expressed by patients and caregivers

The first five most frequent QoL domains expressed by the patients were strictly identical to the first five most frequent QoL domains expressed by the caregivers. Among these five domains, the Family domain was the most expressed QoL domain (expressed by 90% of the patients and 88% of the caregivers). For the 167 dyads for which the family domain was expressed both by the patient and the caregiver, while the attributed weight to the family domain did not differ between the patients and the caregivers, the family score was significantly lower for caregivers than for the patients. Health, Leisures, and Social life were the 3 most expressed QoL domains by the patients and the caregivers, in the second, third, and fourth positions, respectively. For dyads for which Health and Leisure domains were common expressed domains by the patient and the caregiver, the scores did not differ between the patients and the caregivers. However, the weight of the importance given to these respective domains differed: the weight of Health domain was significantly higher for the caregivers than for the patients, and the weight of Leisure score was significantly higher for the patients than for the caregivers. The Occupational issues domain was the fifth domain expressed by the patients and the caregivers, but was most frequently expressed by the caregivers (for 34% of caregivers compared to 27% of patients). Within the dyads including patients and caregivers mentioning the Occupational issues domain, the QoL score was significantly better for the caregivers than for the patients but the weight assigned to the domain was not different between the two members of the dyad.

The caregivers (25%) most frequently mentioned the Love life domain compared to the patients (16%). The 2 members of a same dyad were quite consistent for the following domains: 81% of the dyads, patient and caregiver simultaneously expressed Family, while Love life and Financial issues were not expressed by two thirds of the patient-caregiver dyads, and Environment and Psychological well-being were not expressed by 56% of the patient-caregiver dyads. The index did not differ between patients and caregivers within the same dyad.

**Fig. 1:**
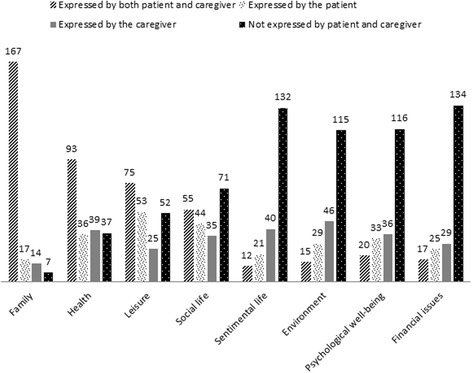
Proportions of expressed SEIQoL domains by the patient-caregiver dyads

The details of the range, the score, and the attributed weight to each of each expressed domains by patients and caregivers are provided in Table 2. For dyads with patient and caregiver expressing common domains, the differences between scores and attributed weight to each domains expressed are detailed in Table 3. Figure 1 illustrates each expressed domains, considering that the domain has been mentioned according to: a) the 2 members of the dyad, b) only the patient within the dyad, c) only the caregiver within the dyad, and d) neither the patient nor the caregiver within the dyad.

**Table 2:** Ranges, scores, and attributed weights of expressed SEIQoL domains by patients and caregivers

	N		Range		Score^a^		Weigh^b^	
	Patients	Caregivers	Patients	Caregivers	Patients	Caregivers	Patients	Caregivers
Domains	*N* = 205	*N* = 205	*N* = 205	*N* = 205	*N* = 205	*N* = 205	*N* = 205	*N* = 205
	N (%)	N (%)	1–10	1–10	(0–100)	(0–100)	(0–100)	(0–100)
Family	184	181	1	1	87,17 ± 17,65	81,64 ± 19,21	40,74 ± 18,19	40,74 ± 20,91
Health	129	132	2	2	55,96 ± 25,57	56,17 ± 25,67	34,57 ± 16,28	37,16 ± 19,43
Leisures	128	100	3	3	51,41 ± 32,64	58,47 ± 28,21	16,24 ± 12,27	14,65 ± 11,43
Social life	99	90	4	4	82,74 ± 17,92	76,96 ± 23,00	17,71 ± 9,84	17,73 ± 12,09
Occupational issues	55	70	5	5	53,56 ± 32,19	75,42 ± 23,04	14,44 ± 10,42	15,53 ± 9,49
Psychological well-being	53	56	6	7	70,63 ± 23,27	69,37 ± 28,93	19,81 ± 13,18	16,98 ± 12,60
Environment	44	61	7	6	64,72 ± 29,98	70,31 ± 26,05	15,3 ± 14,26	11,90 ± 7,14
Financial issues	42	46	8	9	51,35 ± 24,48	62,02 ± 23,89	12,17 ± 6,61	10,43 ± 8,67
Love life	33	52	9	8	80,27 ± 28,60	71,71 ± 24,77	31,97 ± 14,24	28,65 ± 17,93
Autonomy	21	7	10	10	52,41 ± 28,51	61,42 ± 21,15	21 ± 13,13	13,29 ± 8,56

^a^from 0 (lower) to 100 (higher) level of quality of life

^b^from 0 (lower) to 100 (higher) weight assigned to the specific domain

M ± SD: mean ± standard deviation

**Table 3:** Common domains expressed by both patient and caregiver within the dyad

Domains	N	Scores’ difference^a^	*p*	Weighs’ difference ^b^	*p*
Family	167	4.81	**0.002**	0.33	0.918
Health	93	1.6	0.660	4.86	**0.030**
Leisures	75	5.9	0.099	3.34	**0.042**
Social life	55	0.59	0.939	0.80	0.730
Occupational issues	25	19.71	**0.030**	4.50	0.317
Psychological well-being	20	7.96	0.421	7.05	**0.039**
Environment	15	6.32	0.555	4.40	0.486
Financial issues	17	5.11	0.793	1.06	0.594
Love life	12	14.5	0.176	1.00	0.929
Autonomy	2		-	-	-
Index	205	1.72	0.424		

^a^scores’ difference between the patient’ score and the caregiver’ score within a same dyad (absolute value)

^b^weights’ difference between the patient’ weight and the caregiver’ weight within a same dyad (absolute value)

Bold values: *p* < 0.05

### Relations between SEIQoL and dimensions scores of standardized QoL questionnaires

#### Condition-specific QoL questionnaires: EORTC QLQ-C30 and CarGOQoL

For patients, the SEIQoL index was significantly correlated with all the functioning scores of EORTC QLQ-C30 (R from 0.15 to 0.27), except the physical functioning score, and with only 3 of 9 symptom scores (R from -0.18 to -0.23): fatigue, nausea, and pain. The SEIQoL index score significantly correlated with 5 of the 10 dimension scores (psychological well-being, burden, finances, physical well-being, and private life, R from 0.18 to 0.31) and the index of CarGOQoL (*R* = 0.40). All the details are provided in Table 4.

**Table 4:** Correlations between SEIQoL index and dimensions scores of standardized QoL questionnaires

	Patients’ SEIQoL index^a^			Caregivers’ SEIQoL index^a^	
Patient’QoL	R	p	Caregiver’s QoL	R	p
SF-36 (generic)			SF-36 (generic)		
Physical Functioning^a^	0.074	0.314	Physical Functioning^a^	**0.172**	**0.028**
Social Functioning^a^	**0.257**	**<0.001**	Social Functioning^a^	**0.305**	**<0.001**
Role Limitation physical^a^	0.143	0.051	Role Limitation physical^a^	**0.244**	**0.002**
Role Limitation emotional^a^	0.075	0.322	Role Limitation emotional^a^	**0.253**	**0.001**
Mental Health^a^	**0.242**	**0.001**	Mental Health^a^	**0.242**	**0.002**
Vitality^a^	**0.26**	**<0.001**	Vitality^a^	**0.273**	**<0.001**
Bodily Pain^a^	0.136	0.062	Bodily Pain^a^	**0.234**	**0.003**
General Health^a^	**0.257**	**<0.001**	General Health^a^	**0.254**	**0.001**
PCS^a^	0.079	0.304	PCS^a^	0.158	0.052
MCS^a^	**0.245**	**0.001**	MCS^a^	**0.258**	**0.001**
QLQ-C30 (specific)			CARGOQOL (specific)		
Global health^a^	**0.27**	**<0.001**	Psychological well-being^a^	**0.262**	**0.001**
Physical Functioning^a^	0.131	0.076	Burden^a^	**0.313**	**<0.001**
Role Functioning^a^	**0.248**	**0.001**	Relationship with health care^a^	0.127	0.118
Emotional Functioning^a^	**0.266**	**<0.001**	Administration and finances^a^	**0.182**	**0.023**
Cognitive Functioning^a^	**0.152**	**0.041**	Coping^a^	0.105	0.188
Social Functioning^a^	**0.184**	**0.013**	Physical well-being^a^	**0.24**	**0.002**
Fatigue^b^	**-0.232**	**0.002**	Self-esteem^a^	0.053	0.51
Nausea^b^	**-0.209**	**0.005**	Leisures Time^a^	0.132	0.096
Pain^b^	**-0.175**	**0.019**	Social Support^a^	0.094	0.242
Dyspnoea^b^	-0.135	0.071	Private life^a^	**0.237**	**0.004**
Insomnia^b^	-0.144	0.057	Index CarGOQoL^a^	**0.402**	**<0.001**
Appetite^b^	-0.146	0.051			
Constipation^b^	-0.062	0.402			
Diarrhoea^b^	-0.045	0.560			
Financial difficulties^b^	0.021	0.781			

R Spearman correlation coefficient (values between +1 and -1; the sign (+/-) of the correlation coefficient indicates the direction of the correlation) [32]

^a^higher score, higher QoL level

**higher score, lower QoL level

Bold values: *p* < 0.05

#### Generic health-related QoL questionnaire: SF-36

For caregivers, the SEIQoL index was positively but moderately correlated with the mental composite score and 8 dimension scores of the generic QoL questionnaire, the SF-36. The range of significant correlation coefficients was 0.17 to 0.31. For patients, the SEIQoL index significantly correlated with the mental composite score and 4 dimensions score, including social- and mental-like dimensions of SF-36 (social functioning, mental health, vitality, and general health). The coefficients ranged from 0.24 to 0.26. All the details are provided in Table 4.

## Discussion

The quality of life of cancer patients and their caregivers, assessed with QoL standardized questionnaires, has been extensively described in the literature. However, to our knowledge, the present study is the first to simultaneously assess the QoL of a large sample of cancer patients and their caregivers using an individualized approach based on the use of the SEIQoL questionnaire. Despite the individual approach d when describing the SEIQoL-DW, most published reports present results based on the quantitative parts of the instrument. The present study provided, for the first time, QoL domains spontaneously expressed by both cancer patients and their caregivers.

The first interesting finding is individualized measures were moderately correlated with other standardized QoL measures, highlighting that generic, specific and individualized instruments may explore different facets of the self-perceived quality of life [2, 3, 7, 23]. One explanation may be that these instruments may reflect the different conceptual bases of these measures. Unlike traditional QoL instruments, often focusing on QoL limitations inflicted by disease or treatment, SEIQoL may reflect the capacity of a patient to appreciate and value important areas in life, despite health problems. The standardized instruments not only measure pre-defined domains, but also focus more on the symptoms and disease and less on overall QoL of life issues and even positive life aspects. Previous authors have defined the SEIQoL as a person-centered measure, not a health-based measure [24]; spontaneously defining their personal cues and pondering personal priorities should help the individual to clear his/her concept of QoL and provide a rating in a realistic and valid manner [7]. Some limitations of the SEIQoL have previously been described; these include the arbitrary aspect of the nomination of the cues (raw cues grouped in aggregated cues by experts [25]; the multiple administration modalities (individual semi-structured face to face interviews, group settings, telephone interviews, self-administration, computer version…), which may compromise the direct comparability between studies and the heterogeneity of weighting procedure [3]. Thus, this measure should provide an interesting picture in which the individuals take ownership of the QoL concept.

The second interesting finding of this study is that the nature and the frequency of domains spontaneously expressed by a sample of cancer patient was similar to the domains spontaneous expressed by their caregivers. Family was the most frequently expressed domain by both cancer patients and their caregivers, with patients slightly weighing more importance than their caregivers. Family, followed by health and leisure, appears as a common base for various populations that exactly mentioned them in the same sequence: amyotrophic lateral sclerosis patients [25], multiple sclerosis patients [12], cardiac patients [26], or patients undergoing stem cell transplantation [7], or patients with other type of cancer [27]. Among the top 5 domains, the social dimension was the most represented dimension, including family relations, leisure aspect, social life, and even occupational issues. Patients reported lower QoL levels for occupational and financial issues than the caregivers; it is now well-documented that a high proportion of cancer patients become unemployed during the course of the disease (either by job loss or quitting work) resulting to a more difficult financial situation [28]. Autonomy and psychological well-being were less mentioned as independent domain, partially reflecting that the use of the general term of health includes these notions. Other domains were not mentioned, such as spirituality, or less often mentioned, such as financial issues or love life. Some assumptions may be stated. First, these domains strongly depend on socio-cultural and economic context [29]. Second, it is likely that patients and caregivers assume with more difficulties to spontaneously mention these aspects of their life as secondary compared with noble domains, such as ‘family’. Thus, predetermined questionnaires may capture aspects considered as important by the individuals in their daily life but treated with less priority than the disease and treatment importance. Because patients were early in the course of their cancers, future research will benefit from a new focus on the interactions between patients and their relatives later in the course of disease.

The clinical utility of the QoL assessment implementation in clinical practice using this type of measure should be discussed. Individualized measures allow capturing various aspects of QoL, taking into account the uniqueness of the human experience [2, 30]. Wettergren and colleagues recommended the use of these measures in clinical practice to take advantage of the patient’s resources (for example, coping strategies, response shift, and resilience) and act on the problems of greatest importance to the patient. The communication between individuals and therapists should be improved and care could focus on strengthening the patient’s resources and addressing perceived obstacles [31].

## Conclusion

The presents study highlighted additional elements confirming the need to explore QoL using a combination of various questionnaires, associating individualized questionnaires and standardized (generic and/or) questionnaires. Individualized measures enabled individuals to spontaneously express important and non-predefined domains and standardized questionnaires facilitated the expression of self-perception on domains considered secondary.

## Abbreviations

CarGOQoL: CareGiver oncology quality of Life questionnaire
EORTC: European organization for research and treatment of cancer
QoL: Quality of life
SEIQoL: Schedule for the evaluation of individual QoL
SEIQoL-DW: Schedule for the evaluation of individual QoL - direct weighting
SF-36: Short form 36
SF36-MCS: SF36 Mental composite score
SF36-PCS: SF36 Physical composite score
